# Estimating genome-wide off-target effects for pyrrole-imidazole polyamide binding by a pathway-based expression profiling approach

**DOI:** 10.1371/journal.pone.0215247

**Published:** 2019-04-09

**Authors:** Jason Lin, Sakthisri Krishnamurthy, Hiroyuki Yoda, Yoshinao Shinozaki, Takayoshi Watanabe, Nobuko Koshikawa, Atsushi Takatori, Paul Horton, Hiroki Nagase

**Affiliations:** 1 Laboratory of Cancer Genetics, Chiba Cancer Center Research Institute, Chuo-ku, Chiba, Japan; 2 Artificial Intelligence Research Center, National Institute of Advanced Industrial Science and Technology (AIST), Koto-ku, Tokyo, Japan; 3 Laboratory of Innovative Cancer Therapeutics, Chiba Cancer Center Research Institute, Chuo-ku, Chiba, Japan; 4 Department of Computer Science and Information Engineering, National Cheng Kung University, Tainan, Taiwan; 5 Institute of Medical Informatics, National Cheng Kung University, Tainan, Taiwan; University of Mysore, INDIA

## Abstract

In the search for new pharmaceutical leads, especially with DNA-binding molecules or genome editing methods, the issue of side and off-target effects have always been thorny in nature. A particular case is the investigation into the off-target effects of *N*-methylpyrrole-*N*-methylimidazole polyamides, a naturally inspired class of DNA binders with strong affinity to the minor-groove and sequence specificity, but at < 20 bases, their relatively short motifs also insinuate the possibility of non-unique genomic binding. Binding at non-intended loci potentially lead to the rise of off-target effects, issues that very few approaches are able to address to-date. We here report an analytical method to infer off-target binding, via expression profiling, based on probing the relative impact to various biochemical pathways; we also proposed an accompanying side effect prediction engine for the systematic screening of candidate polyamides. This method marks the first attempt in PI polyamide research to identify elements in biochemical pathways that are sensitive to the treatment of a candidate polyamide as an approach to infer possible off-target effects. Expression changes were then considered to assess possible outward phenotypic changes, manifested as side effects, should the same PI polyamide candidate be administered clinically. We validated some of these effects with a series of animal experiments, and found agreeable corroboration in certain side effects, such as changes in aspartate transaminase levels in ICR and nude mice post-administration.

## Introduction

*N*-methylpyrrole-*N*-methylimidazole (PI) polyamides have been frequently discussed as viable alternatives for “undruggable” oncological targets otherwise inaccessible at the protein level, such as *KRAS* [1–3]. Their ability to interact with the DNA minor-groove binders with unrivaled sequence recognition translates to their distinctive ability to disrupt transcription, and proper functionalization with histone modifiers or alkylating agents transform these molecules to programmable epigenetic switches and anticancer agents *in vivo* [4–10]. A troubling issue for PI polyamides, however, is their relatively short motifs, leading to multiple genomic binding sites; lengthening a PI polyamide beyond its typical working range of 8–10 bases will reduce the number of genomic binding sites at the penalty of reducing its chemical affinity to the DNA minor groove due to increased structural rigidity [11, 12]; intriguingly, most PI polyamide, despite the presence of multiple binding sites, led to little toxicity [13–15]. It would therefore imply that nonunique binding typically did not lead to ill biological effects, further complicating our understanding on the subject matter. Nevertheless, to improve the clinical prospects of PI polyamides, the evaluation of these so-called “off-target effects” would be unavoidable in a similar manner as genome editing methods such as CRISPR/Cas. Yet, even before we could approach the problem, we were met with a difficult question: for PI polyamides, how would one define the effect of off-target binding for PI polyamides?

In the most general sense, binding to any genomic location other than its intended site is technically “off-target.” Several studies have elected to use next-generation sequencing (NGS) methods such as Chem-seq [16–18] to approach the question of off-target binding. While Chem-seq do provide the means of identifying binding sites in the genomic space, in addition to functionalizing a candidate PI polyamide with an affinity tag for enrichment, the method still requires the coupling of expression profiling to characterize the effect of genomic binding. Moreover, binding at an unintended site in certain situations may be more or less inconsequential; for instance, binding in genes downstream of the intended target may do very little since the process is already disrupted. To understand the effect of off-target binding, therefore, would necessitate one to perform additional analysis and process the expression profiling data beyond the conventional before/after fold-change calculations to elucidate or distinguish these effects.

Common knowledge suggest that chemical stimuli can environmental or oxidative stress homeostasis, leading to the alteration of expression profiles as a response to those perturbations. Consequently, while some genes are certainly to be downregulated upon the administration of a PI polyamide, it is entirely possible that those changes are as part of the homeostatic responses rather than “off-target” binding; how does one differentiate “true” off-target binding then? We can reformulate the question as follows: if a polyamide suppresses its primary target by some extent, is that suppression significant when compared to non-targets? Now, the natural response would be to compare changes to the primary target with some measures of significance testing with the rest of the targets in a background population; this nonetheless gets complicated, as gene expressions are also affected by innate feedback, homeostasis, regulatory mechanisms and complex secondary effects *in vivo*. By recognizing that most genes participate in various pathways that lead to phenotypic changes, these pathways can more natural units of analysis to evaluate off-target binding. The null hypothesis then is whether the effect onto “on-target” pathways differs from other “off-target” pathways. To evaluate these changes, NGS methods are also no longer required; expression microarrays in this regard are viable alternatives in exploring a potential candidate PI polyamide’s off-target effects, since one can simultaneously monitor changes in the genome at a reasonable cost through with nearly 50,000 data points per run.

With this testable hypothesis in mind, we devised a computational method to evaluate the relative impact to unintended pathways (“background”), compared to pathways with target participation (“foreground”). By evaluating the impact to a pathway as a mean change of all participating genes, we could search the background to identify genes relatively downregulated compared to the intended target to estimate the extent of erroneous binding. We here propose the use of expression microarrays to evaluate the effect of off-target binding and possible side effects via a pathway-based approach. The effectiveness of our analysis is further validated in mouse experiments. This is the first report of a method specifically aimed to address the complicated issue of off-target binding for PI polyamides, and hopefully these algorithms and methodologies may help accelerate the much-needed effort in motif-specific DNA binders in the field of chemical biology and precision medicine.

## Materials and methods

### Summary of polyamide synthesis

Polyamides **1**–**4** were synthesized piecewise following a Fmoc-based solid-phase peptide synthesis protocol on a PSSM-8 peptide synthesizer (Shimadzu) with a custom operating system and functionalized by indole-*seco*-CBI [19]. Purification of the product was performed using high-performance liquid chromatography system on a LC-20 (Shimadzu) using a 10 mm × 150 mm Gemini-NX3u 5-ODS-H reverse-phase column (Phenomenex). Separation was achieved with a linear gradient of acetonitrile in 0.1% acetic acid in Milli-Q water (typically 0–100%) at a flow rate of 10 ml *m*^-1^ with detection at 310 nm. Collected fractions were analyzed by a LC-MS2020 LC-MS system (Shimadzu). Conjugation of indole-*seco*-CBI was performed by a reaction of respective PIP backbone with NH_2_-indole-*seco*-CBI in *N*-methylpyrrolidone in the presence of 1-ethyl-3-(3-dimethylaminopropyl)carbodiimidyl hydrochloride. Reaction mixtures were then purified by liquid chromatography with a linear gradient of in 0.1% acetic acid in water (typically 30–75%), and the final products were subsequently lyophilized. Mass information (ESI-MS): polyamide **1**, C_100_H_110_ClN_34_O_18_S, *m/z* calcd [M+2H]^2+^ 1071.95, found 1072.00; [M+3H]^3+^ 714.94, found 715.25; polyamide **2**, C_89_H_92_ClN_31_O_16_, *m/z* calcd [M+H]^+^ 1886.70, found 1886.50; calcd [M+2H]^2+^ 943.86, found 944.05; calcd [M+3H]^3+^ 629.57, found 629.45; polyamide **3**, C_88_H_91_ClN_32_O_16_, *m/z* calcd [M+H]^+^ 1888.32, found 1888.95; polyamide **4**, C_89_H_92_ClN_31_O_16_, *m/z* calcd [M+2H]^2+^ 943.86, found 944.15; calcd [M+3H]^3+^ 629.57, found 630.10.

### Cell cultures

Cells were maintained in a humidified environment at 37°C and 5% CO_2_ in their respective media and nutrient supplements (S1 Table) in addition to 1% penicillin/streptomycin. Reagents used for cultures were purchased from Gibco.

### Microarray data processing

10^4^–10^5^ cells per condition were plated in a 6-well microtiter plate for overnight attachment prior to treatment of candidate polyamides or 0.05% dimethyl sulfoxide (DMSO). After RNA extraction with RNeasy Plus Mini Kit (Qiagen), replicated samples (2 × 2 for polyamides **1** and **2**; 3 technical replicates for **3**) were labelled with RNA Spike-In Kit and analyzed on SurePrint G3 Human GE 8x60K V2 microarrays per Agilent Technologies’ recommendations. Data acquisition was performed on an Agilent SureScan microarray scanner and analysis were completed with custom R codes utilizing the *limma* package [20]. Our procedure automatically searched for a suitable offset to correct for background effects and normalize replicate between arrays and determined expressions by empirical Bayes estimations in terms of fold changes from the difference between DMSO and each PI polyamide treatment. Spots with matching RefSeq mRNA & ncRNA identifiers, as well as Ensembl accession codes if applicable, were filtered. For the purpose of comparison, genes with promoter region (defined as 1000 bp upstream of transcription start site) binding were also annotated with corresponding mRNA expressions. Tissue-specific expression changes (as defined by the logarithm of mean tumor expressions compared to normal tissue expressions) from GENT [21] were extracted for the genes considered to be “on-target” to use as weights in the computation.

### Pathway analysis

Gene-pathway data were retrieved from the Kyoto Encyclopedia of Genes and Genomes (KEGG) [22]. After selecting a cell line as control (in the case of polyamide **2**, SiHA), expression data in the form of a 6-column table detailing the chromosomal position of the gene, a corresponding RefSeq symbol and accession number followed by the expression in terms of logFC) were recomputed to determine the relative change in expression (RFC) across the test and control cell lines. These RFC’s were then mapped to a specific pathway (PI3K-Akt signaling pathway, hsa04151) based on pathway-gene information retrieved from KEGG; each clustered gene on the KEGG pathway map was then assigned a color based on their respective RFC; for those with RFC < -0.5, red; RFC > 0.5, green; orange otherwise. Binding site information was obtained from either Chem-seq experiment (polyamide **1**) or based on searching the number of motif occurrences in the hg19 reference genome according to the order of methylpyrrole and methylimidazole pairings. The core pathway (genes immediately upstream of *PIK3CA* and downstream of Akt) was visualized using PathVisio [23].

### Side effect prediction

Drug-adverse reaction data were obtained from DrugBank and SIDER following previously published procedures [24, 25] to generate a final matrix of drug-adverse reaction matrix. Gene expression datasets containing the selected drug candidates found in the matrix were manually retrieved from the GEO database (S2 Table) and those expressions were processed as training sets as predictors for each of the adverse reactions included in the matrix. Side effects occurring in fewer than 10 of the training set drugs were omitted. Expressions for primary target genes (e.g. *KRAS* for **1** and *PIK3CA* for **2**) were numerically scaled with recorded tumor/normal expression ratios from GENT in their respective cancer origins prior to side effect prediction (see S3 Table for an example for *PIK3CA*). Random forest via the R *ranger* implementation with 1000 trees per ensemble was used for data training to generate a side effect prediction model for polyamides **1**–**3**. Codes used for pathway analysis and building side effect prediction models were organized into *pipoft*, an R package available on GitHub (jlincbio/pipoft).

### Animal experiments

Approval from the animal care and ethics committee of Chiba Cancer Center Research Institute were obtained and experiments were performed following strict guidelines to minimize animal suffering. Mice were housed in a designated restricted-access facility staffed by independent Japan Animal Care (JAC) personnel and provided with sterile water and CRF-1 Certified Diet (Oriental Yeast) feed pellets over the course of experimentation. Physical examinations and non-interactive visual checks were routinely performed to ensure subjects’ well-being until sacrifice by cervical dislocation. Collection of blood specimens were performed under anesthesia by minute amounts of ether, to effect. Invasive procedures and sacrifice were performed by a licensed veterinarian (A.T.). Female Balb/c nude mice (Charles River Laboratories) were subcutaneously inoculated with 10^6^ ME-180 cervical cancer cells. Upon reaching a critical tumor size of ~ 50–70 mm^3^, the mice were intraperitoneally injected with DMSO (*n* = 4) or 3 mg/kg·week polyamide **2** (*n* = 5) and sacrificed after the tumors reached 2 × 10^3^ mm^3^. For polyamide **1**, sera were collected from mice treated with DMSO, 0.3 or 3 mg/kg of polyamide **1** at 3 and 36 days. To evaluate the effect of 10 mg/kg polyamide **4**, we first performed a pilot experiment with two C57BL/6J mice to assess the need for an additional cohort of 4 × 2 ICR mice (Charles River Laboratories) for monitoring changes in appetite as well as AST, ALT, blood glucose levels. Mice were housed in individual cages so the amount of feed remaining in the food compartment could be measured in 24 h periods during the course of polyamide **4** treatment. The test and control groups were injected with a dose of 10 mg/kg polyamide **4** or DMSO in sterile phosphate-buffered saline solutions once a week. After sacrifice on day 16, liver tissues (C57BL/6J mice, CLEA Japan) and serum samples (ICR mice) were collected for histological examination and biochemistry panels. 150 μL of blood per mouse was incubated with 10 μL coagulant (Wako) at room temperature for 0.5 h before centrifugation at 16 × 10^3^ rpm for 15 m for serum collection. Samples were stored at –80°C until analysis. Quantification of AST, ALT, CRE and blood glucose levels were performed by Starlight Biotech Inc. (Tokyo, Japan) or the Japan Mouse Clinic at RIKEN (Tsukuba, Japan) in the case of the pilot experiment with polyamide **4**.

### Cytotoxicity assays and correlation to *GNA14*

Cells (S4 Table) were seeded at a final density of 3 × 10^3^ cells in a 96-well microtiter plate (Nunc) and incubated overnight for attachment. Upon treatments of various concentrations of polyamide **4** or 0.125% DMSO for 48 h, 10 μL WST-8 reagent (Dojindo) was added to each sample and incubated at 37°C for 2 h to assess the rate of cell proliferation. Absorbances were measured at 450 nm on a Corona MTP-310 microplate reader, with IC_50_ values calculated using GraphPad Prizm. *GNA14* expressions were obtained from compiled U133A values in GENT [21].

### Statistical analysis

Two sample *t*-tests, unless otherwise specified, were used to assess the null hypothesis that there were no differences in the sample means between gene expressions or marker levels. For gene expressions, we assessed significance against a predefined *α*-level of 0.05; for the mouse experiments, as sample sizes were *n* < 10 per experiment, we adopted the one-tailed *α*-level of 0.10, such that fewer than one out of the entire cohort may be a false positive at any time. Power analyses were performed with the *pwr* package in R [26, 27] to ensure that the adaptation of the new alpha level did not impact the assessment of significance (criteria: *p* < 0.10, statistical power > 90% and within 15% of the same metric under *p* < 0.05).

## Results and discussion

### Pathway-based approach to evaluating off-target effects

**Fig 1:**
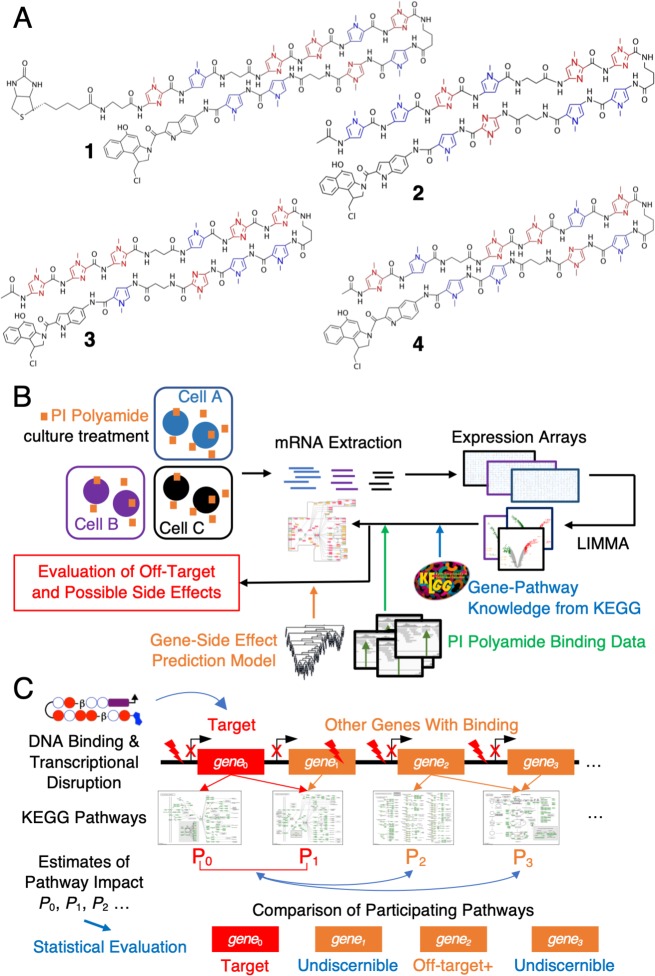
Design of a pathway-based method to evaluate off-target effects for PI polyamides. (A) Structure of candidate PI polyamides: **1**, designed to target G12D/V KRAS mutants; **2**, a polyamide seeking to bind E545K PIK3CA mutation; **3**, a polyamide meant to suppress *MYCN* amplifications; **4**, unbiotinylated version of polyamide **1** used for *in vivo* validation experiments. *N*-methylpyrrole subunits, blue; *N*-methylimidazole subunits, red. (B) Scheme of automated systematic Analysis; (C) concept of pathway-based evaluation.

We utilized PI polyamides **1–3** (Fig 1A) targeting *KRAS* G12D/V mutations, *PIK3CA* E545K mutation and a third candidate which sought to suppress *MYCN* amplification as its mode of action [28, 29] to determine whether the newly formed hypothesis sufficiently addressed the question of determining the extent of off-target effects at the pathway level; polyamide **4**, an additional unbiotinylated version of polyamide **1**, was used for a follow-up validation experiment *in vivo*. All three polyamides were functionalized with indole-*seco*-CBI, an alkylating moiety; while polyamide **1** had an additional biotinyl moiety as it was originally developed for NGS applications, we previously [16] found no discernible differences compared to the non-biotinylated counterpart in microarray studies.

After administrating these polyamides to their respective target cell lines (Fig 1B), we determined changes in expression (logFC) systematically and linked gene-pathway information available in KEGG to calculate the relative impact for all genes participating in the catalogued pathways. We used the relative percentile rank of logFC and measured their deviations from the median to provide a method to evaluate whether these differences were statistically meaningful. The incorporation of binding information (either from NGS results such as Chem-seq or by motif-based computational estimation), allowed us to determine the extent of “off-target” binding (Fig 1C). This procedure allowed us to separate severely affected genes from “undiscernible” genes that might have been affected by PI polyamide treatment but did not overall behave much differently from other members in the same pathway.

**Fig 2:**
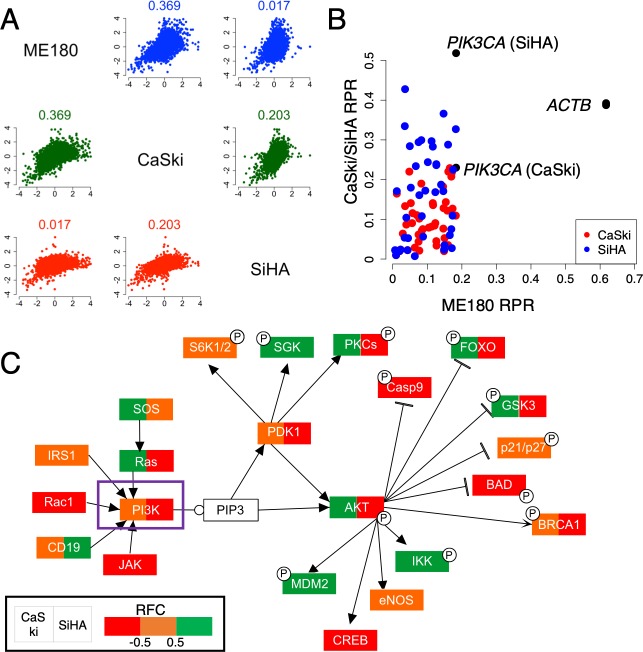
Off-target evaluation of polyamide 2. (A) Correlations between changes in expression (logFC) after treatment of 10 nM of polyamide **2** against DMSO across ME180, CaSki and SiHA cells; numerical values above each pairing indicate the pairwise Spearman’s rank correlation coefficient. (B) Distribution of the relative percentile rank (RPR) for on-target and off-target genes expressed as ME180/CaSki (red) or SiHA (blue) pairings. *PIK3CA* (on-target) or *ACTB* (control) is expressed in black. (C) Differential comparison of changes in expression across different cell lines in the PI3K-Akt pathway (KEGG accession: hsa04151), with ME180 as the baseline; colors indicate the level of RFC (< -0.5, red; > 0.5, green), with divided boxes indicating nonuniform RFC behavior between CaSki/ME180 (left) and SiHA/ME180 (right) pairings. PIP3, phosphatidylinositol-3,4,5-triphosphate. Full pathway illustrations can be found as S1 and S2 Figs.

This method allowed us to estimate the relative effect of our candidate PI polyamides to their respective targets (Table 1). Polyamide **2**, for instance, was estimated to have a larger number of off-target genes in SiHA, a cervical cancer cell line expressing wild-type *PIK3CA*; while this polyamide was capable of disrupting mutant *PIK3CA* preferentially, the cytotoxicity was still in the nanomolar range, the high number of off-target genes would provide an explanation for this observation. We found expressions to correlate roughly in mutant *PIK3CA* cell lines (ME180 and CaSki, Fig 2A), and the three cell lines shared a number of identical genes deemed “off-target” (Fig 2B). To further clarify how “on-target” polyamide **2** was, we expanded the algorithm to consider the relative differences between SiHA and other mutant *PIK3CA* cell lines. Since *PIK3CA* had been frequently asserted to be the primary driver gene in cervical cancer, assuming that most other mutations manifested little effect on the cancer phenotypes, we used SiHA as the “baseline” and computed the extent of which the other two cell lines deviated in terms of expressions in the PI3K-Akt signaling pathway. We saw a downward shift of this relative expression difference for *PIK3CA* as well as several downstream elements (Fig 2C and S1 and S2 Figs), for instance *AKT*, *PTEN* and p53 in the case of ME180 cells (by comparing SiHA/ME180 and CaSki/ME180 pairings). Several downstream elements of *PIK3CA* were invariant in their responses in SiHA and CaSki cells, suggesting that they were little affected by the binding of polyamide **2**, allowing us to disregard quantitatively the effects to those elements and their subsequently participating pathways.

**Table 1:** Assessment of off-target binding of polyamides 1–3.

Polyamide	Cell Line	Target RPR	Off-Target Genes
1	LS180	0.3084	13
1	SW480	0.2962	59
2	ME180	0.1838	54
2	CaSki	0.2310	62
2	SiHA	0.5188	194
3	CHP134	0.3744	509*
3	KELLY	0.4384	652*
3	SK-N-AS	0.0493	56*
3	MCI C6	0.3596	519*

RPR, relative percentile rank; significance level for genes with RPR below primary target: *p* < 0.05

*Significance of off-target genes was not evaluated as there were an insufficient number of KEGG pathways annotated with *MYCN* at the time of analysis.

### Inferencing potential side effects of PI polyamides

**Fig 3:**
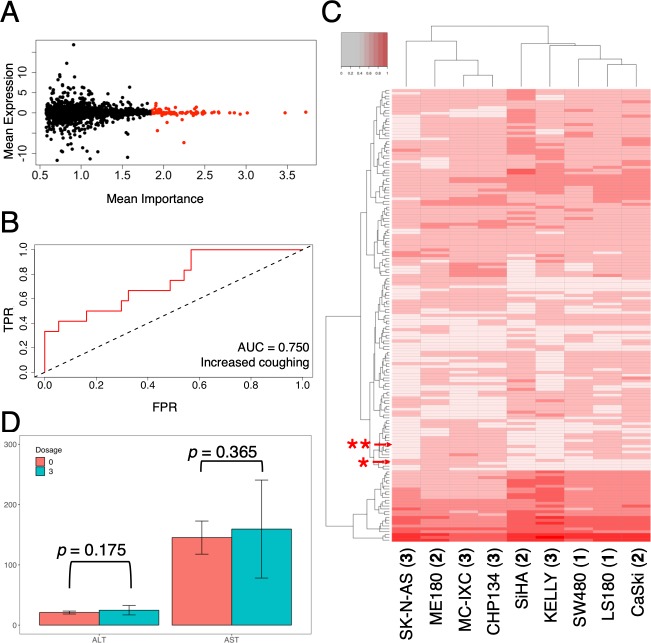
Side effect evaluation of PI polyamides. (A) comparison of gene expressions and its relative importance in the prediction model; vertical axis, Z-normalized mean expressions for a particular gene in training data sets; horizontal axis, importance in terms of mean Gini index; top 100 most important genes were highlighted in red. (B) sample receiver operating characteristic curve for the side effect of increased coughing; *FPR*, false positive rate; *TPR*, true positive rate; *AUC*, area under curve (0.750). (C) Hierarchical clustering of common potential side effects of polyamides 1–3 from expression profiling; Cell lines and the corresponding polyamide are indicated in parentheses; side effects with likelihood scores are indicated in increasing shades of red. Arrows and asterisks indicate AST (*) and ALT (**) increases. (D) AST and ALT levels of ME180 xenograft in Balb/c nude mice after 30–35 d of exposure to 3 mg/kg polyamide **2** (cyan) or DMSO (0 mg/kg; orange). Statistical significance is assessed by two-sample *t*-test; error bars indicate mean ± SD.

We now expand the discussion on off-target binding to understand the potential phenotypic changes that may be more clinically relevant and easier to conceptualize: side effects. The ability to eliminate PI polyamide candidates with more adverse effects *a priori* could beneficially improve the design and testing process and provide a way to monitor animal experiments to track and observe these small but critical shifts in phenotype. While various side-effect prediction methods exist [24, 25, 30, 31], most had been developed with protein-level based inhibitors in mind and focused largely on modelling changes through metabolic networks; since PI polyamides’ primary mode of action was DNA minor-groove binding, we thought that perhaps an approach utilizing gene expression profiling from microarray data would better reflect the direct effect more meaningfully. An expression-based prediction strategy also allowed us to account for the hypothetical possibility that certain gene targets could have a wide range of expression distributions in different tissues by allowing for separate microarray datasets to be evaluated or directly adjusting for expression discrepancies by scaling factors derived from tissue-specific information. We chose to implement this approach by testing different machine-learning methods such as support vector machines and neural networks with a publicly available set of cell-line expression arrays under various drug treatments coupled with available side effect information as training data. Upon testing, we found random forests to be the most robust; the method itself required merely a fraction of computing time compared to neural networks at the current size of drug-adverse reaction arrays and had lower prediction errors compared to support vector machines. Predictions across different cell lines of the same cancer type, i.e. SW480 vs. LS180 of colorectal cancer, or the ME180 vs. CaSki of cervical cancer (S3–S5 Figs) also were more consistently correlated compared to raw expressions. We could also systematically identify key elements leading to certain side effects and evaluate the reliability of our predictions (Fig 3A). The predictor models were sufficiently robust, with a large number of side effects yielding AUCs greater than 0.70 (an example is provided in Fig 3B).

### Side effect predictions with animal experiments

Throughout our testing of the three polyamides, several side effects were common across different cell lines and polyamides (Fig 3C and S5 Table); a large number of the side effects were qualitative (neuropathy, for instance), and barring actual patient-based testing there were few ways to validate these predictions. As the prediction model suggested that polyamide **2** could potentially alter aspartate and alanine transaminase levels (AST and ALT, respectively) among other qualitative side effects, we tried to confirm these quantifiable changes in concurrent *in vivo* experiment with ME180 xenografts in Balb/c nude mice and 3 mg/kg polyamide **2**, a dose an order of magnitude below the estimated maximum tolerated dose (MTD) of 30 mg/kg based on our previous studies of other polyamides. Inspections of AST and ALT levels in these mice revealed increases supporting the likelihood of our hypothesis (Fig 3D; two-sample *t*-test *p* = 0.365 and *p* = 0.175 for AST and ALT, respectively), albeit with the difference in sample means above the threshold for significance. We also evaluated whether to reject the null hypothesis that there was no difference in the sample variance between the control and test groups with two-tailed *F* test (*p* = 0.1056 and *p* = 0.0869 for AST and ALT, respectively).

**Fig 4:**
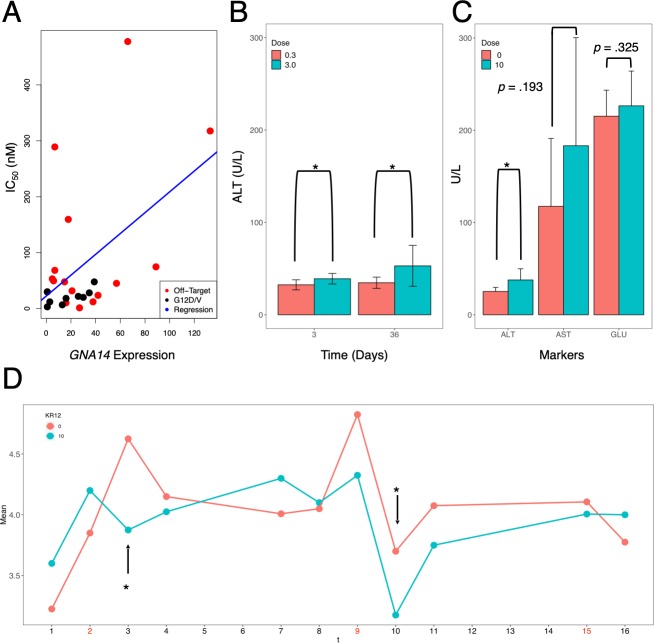
Side effects of polyamides targeting *KRAS* with G12D/V mutations. (A) Correlation of cytotoxicity of polyamide **4** to cell lines of varying KRAS codon 12 mutation status and their respective *GNA14* expression. Cytotoxicity is expressed as the IC_50_ concentration determined by cell proliferation assay; red points indicate cell lines with non G12D/V *KRAS* mutation (“off-target”); black points indicate cell lines with G12D/V *KRAS* mutations. *R*^*2*^ for correlation model [IC_50_] = *a*_*0*_ + *a*_*1*_ [GNA14]: 0.2463; with *a*_1_ = 1.8635 (*p* = 0.0116). For reference, *R*^*2*^ for correlation model [IC_50_] = *a*_*0*_ + *a*_*1*_ [KRAS]: 0.04128, with *a*_*1*_ = -0.0430 (*p* = 0.3300). (B) ALT levels of Balb/c nude mice after 3 or 36 *d* of exposure to polyamide **4**; *n* = 4 per test condition; orange, 0.3 mg/kg polyamide **4**; cyan, 3.0 mg/kg. (C) Serum ALT, AST and glucose levels of ICR mice as well as (D) changes in appetite over time in ICR mice treated with polyamide **4** (*n* = 4 each; 0 mg/kg, orange; 10 mg/kg, cyan); red timepoints in the horizontal axis indicate points of injection. Error bars indicate mean ± SD; statistical significance is assessed by two-sample *t*-test; ‘*’, one-tailed *p* < 0.10. Arrows in (D) indicate days in which treatment of between 0 or 10 mg/kg of polyamide **4** showed statistical significance.

To confirm this prediction in other systems, we then performed a set of similar analyses with polyamide **4** (the unbiotinylated version of **1**) against *KRAS* G12D/V mutations. We first computationally explored potential candidates prone to off-target binding, such as the type and number of off-target genes, as well as their possible connections to the primary target. We found several genes to be affected as a result of off-target binding (S6 Table). These results mirrored some of the previously established links [32–34] between *KRAS* and *EEA1*, an endosomal marker as well as functional changes in *GNA14*. We tested a number of cancer cell lines for their sensitivity to polyamide **4** and were able to observe some correlation to *GNA14* levels (Fig 4A and S4 Table); from this result, certain links likely existed between off-target binding to *GNA14* and the resultant cytotoxic response. With recent reports that *GNA14* mutations could be associated to liver abnormalities such as hepatic small vessel neoplasm [35], we then suspected that the accidental disruption of *GNA14* could lead to, and potentially explain, the manifestation of liver-related effects. As results with C57BL/6J mice over the course of two weeks from a pilot experiment suggested marginal changes in ALT levels (Fig 4B) under the influence of polyamide **4**, we set up a differently structured side effect experiment with a follow-up cohort of ICR mice (*n* = 8) at 10 mg/kg polyamide **4**, a dosage level approximately half the order of magnitude from the MTD. In this experiment, we monitored changes in appetite, a predicted side effect, as well as AST, ALT and blood glucose (GLU) levels. We found AST levels to fluctuate more appreciably compared to ALT or GLU, although changes in ALT level were significant while AST marginal (Fig 4C; GLU, an unpredicted side effect, was determined to be invariant). This trend was markedly consistent in mice treated with different polyamides (Figs 3D, 4B and 4C) as similar to earlier results. As a consequence of the small cohort size (*n* < 10), we decided to assess significance at the alpha level of *p* < 0.10, or at most one per ten subjects could be a false positive; generally, elevating the alpha level had no negative effect on the statistical power during testing. The mice were responsive to polyamide treatment and displayed changes in the distribution of ALT levels in the cohort. Additionally, on days immediately following an injection, mice administered with polyamide **4** tended to exhibit reduced food intake (Fig 4D, black arrows) while changes in body weight were insignificant across the two groups.

These increases echoed earlier findings that certain, but not all, configurations of PI polyamides could induce increases in these aminotransferases [14] when amine substitutions were introduced to the γ-turn subunit. Considering that previous reports found the toxicity to be highly dose-limited and inconclusive on the contribution of polyamide structure on toxicity, an expression profiling-based approach would be more reliable for estimating the side effect of a polyamide due to potential off-target effects. Increases in blood creatinine level, a side effect predicted not to occur with polyamide **2**, were found to be negative (~0.1–0.2 mg/dl), once again placing the noted aminotransferase increases in focus. ALT and AST levels recorded in these subjects tended to fall within the normal range of 25–60 U/L and ~ 50–100 U/L, respectively [36], suggesting that liver function was most likely also normal, well in corroboration with a previous examination of liver tissues in animal subjects [28]. Hierarchical clustering of the side effects additionally suggested no close relations to other liver-related side effects, and even at 10 mg/kg polyamide **4**, elevations in AST and ALT did not correlate with liver damage upon histological examination (S6 Fig). Further model optimizations factoring in considerations such as concentration and dosage lengths could help further differentiate effects driven by the pharmacodynamics of PI polyamides in tumor and normal tissues. While cell-level observations may not fully translate to higher-order animal results, this procedure has the benefit of being able to screen out quickly the less desirable PI polyamide candidates. In lead development, initial decisions to proceed with certain candidates are frequently generated based on cell-level experiments, and our method can be implemented in-line of other experiments with relative ease.

## Conclusion

Most developmental pipelines encounter a similar problem: it is not always certain whether the investment in preclinical studies will attribute to the eventual success for a marketable product. PI polyamides, while highly versatile and by their mechanistic nature tunable, also face this. By evaluating candidate polyamides with expression microarrays and this systematic method can greatly improve the throughput and meaningfully shorten the initial lead selection and optimization period. We understand how changing the structure of PI polyamides may affect its binding affinity, and how these molecules’ relative hydrophobicity can have on its tumor retention [37]; yet we still know very little about how these molecules will influence the outward phenotypes, especially when binding motifs are concerned. The lack of progress in understanding the off-target effect of PI polyamides can limit the growth of PI polyamide research and the translation to bedside applications. With our first report on the subject matter, we hope to facilitate the push of PI polyamides to bedside applications and accelerate the field of precision medicine.

## Supporting information

S1 Fig Full PI3K-Akt pathway for CaSki/ME180 cell lines upon administration of polyamide 2. Levels of relative fold change across cell lines (RFC) as indicated: yellow, *|RFC|* < 0.5; red, *RFC* < -0.5; green, *RFC* > 0.5.
(TIF)

S2 Fig PI3K-Akt pathway for SiHA/ME180 cell lines upon administration of polyamide 2. Levels of relative fold change across cell lines (RFC) as indicated: yellow, *|RFC|* < 0.5; red, *RFC* < -0.5; green, *RFC* > 0.5.
(TIF)

S3 Fig Correlation of expressions and side effect predictions of polyamide 1 in LS180 and SW480 colorectal cancer cells. Above, green: correlation of gene expressions in LS180 (horizontal axis) and SW480 (vertical axis); below, purple: correlation of predicted side effect scores in LS180 (horizontal) and SW480 (vertical).
(TIF)

S4 Fig Correlation of expressions and predicted side effects of polyamide 2 in ME180, CaSki and SiHA cervical cancer cells. Above diagonal, correlations of expressions (green); below diagonal, pairwise correlation of side effect scores (purple). Cell lines are labelled diagonally across the vertical and horizontal axes.
(TIF)

S5 Fig Correlation of expressions and side effect predictions of polyamide 3 in CHP134, KELLY, MC-IXC and SK-N-AS cell lines. Above diagonal, correlations of expressions (green); below diagonal, pairwise correlation of side effect scores (purple). Cell lines are labelled diagonally across the vertical and horizontal axes.
(TIF)

S6 Fig Histological staining of the skin and liver from polyamide-treated C57BL/6J Mice. Left half, skin; right half, liver tissue staining. DMSO (top half) is labeled 0 mg/kg; bottom, polyamide **4** at the dose of 10 mg/kg.
(TIF)

S1 Table List of cells and culture conditions. ATCC, American Type Culture Collection; ECACC, European Collection of Authenticated Cell Cultures; RIKEN, RIKEN BioResource Research Center Cell Bank; NCI, National Cancer Institute Division of Cancer Treatment and Diagnosis Tumor Repository; MEM, Eagle’s Minimum Essential Medium; DMEM, Dulbecco’s Modified Eagle’s Medium; RPMI-1640, Roswell Park Memorial Institute medium 1640; IMDM, Iscove’s Modified Dulbecco’s Medium; FBS, fetal bovine serum; Gln, glutamine (if supplemented).
(PDF)

S2 Table Training expression datasets for the side effect prediction model. (PDF)

S3 Table Relative distribution of *PIK3CA* in various tumor and normal tissues. Values computed from average tissue-type U133 Plus 2 *PIK3CA* expressions in GENT as of August 2018; *logFC* indicates the log ratio of tumor vs. normal expressions for a particular tissue.
(PDF)

S4 Table Summary of cytotoxicity of polyamide 4 and *GNA14* expressions in various cell lines. *KRAS* G12, mutation status for codon 12 in *KRAS*; *GNA14*, expression of *GNA14* in U133A datasets compiled by the Gene Expression Across Normal and Tumor Tissue Database. *Class*, classification of cell lines into “Off-Target” or “G12D/V” in Fig 4A.
(PDF)

S5 Table Common potential side effects of polyamides 1–3 from metabolic gene expression profiling. Predicted scores are on a scale of 0–1; *synd*., syndrome; *inc*., increase; *dec*., decrease; *did*., disorder. The table is illustrated as a heatmap in Fig 3B.
(PDF)

S6 Table Potential off-targets of polyamide 1 in LS180 colorectal cancer cell line. *RPR*, mean relative percentile rank of specified gene in all participating pathways; p_0_, *t*-test *p*-value under H_0_: *RPR* = 0.5000; p_1_, *t*-test *p*-value under H_0_: *RPR*_Off-Target_ = *RPR*_Target_. Significance evaluated against a predefined threshold of *p* < 0.05 (log_10_ *p* < -1.3010); *N/S*, not significant; *N/A*, not evaluated; ^[a]^number of pathways evaluated for *KRAS* (“foreground”): 68; number of background pathways: 244.
(PDF)
