# Supplementary figures and images for: Estimating genome-wide off-target effects for pyrrole-imidazole polyamide binding by a pathway-based expression profiling approach
Source: PLoS One. 2019 Apr 9;14(4):e0215247. doi: 10.1371/journal.pone.0215247 (PMC6456183; doi:10.1371/journal.pone.0215247)

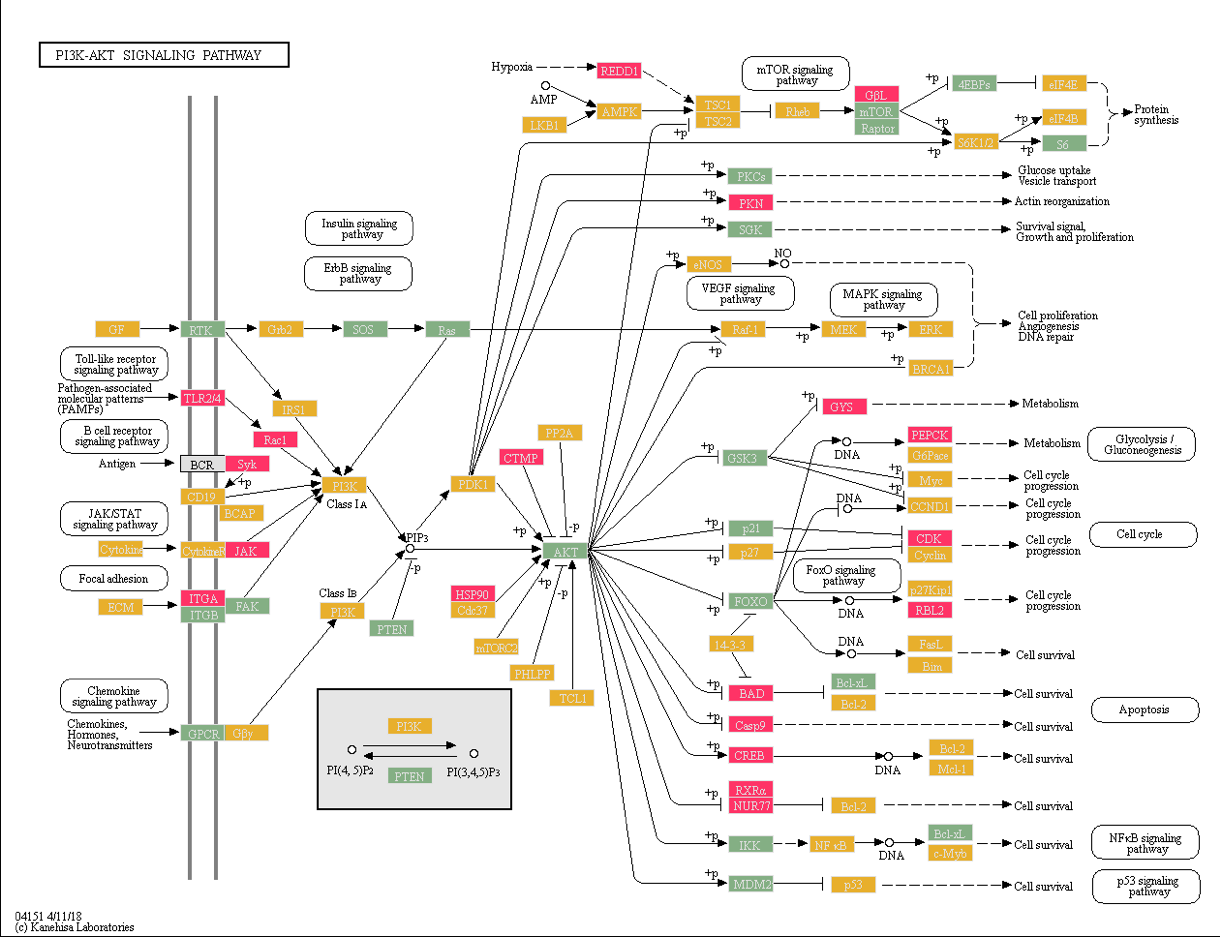

Supplement: S1 Fig — Levels of relative fold change across cell lines (RFC) as indicated: yellow, |RFC| < 0.5; red, RFC < -0.5; green, RFC > 0.5. (TIF) [file pone.0215247.s001.tif]

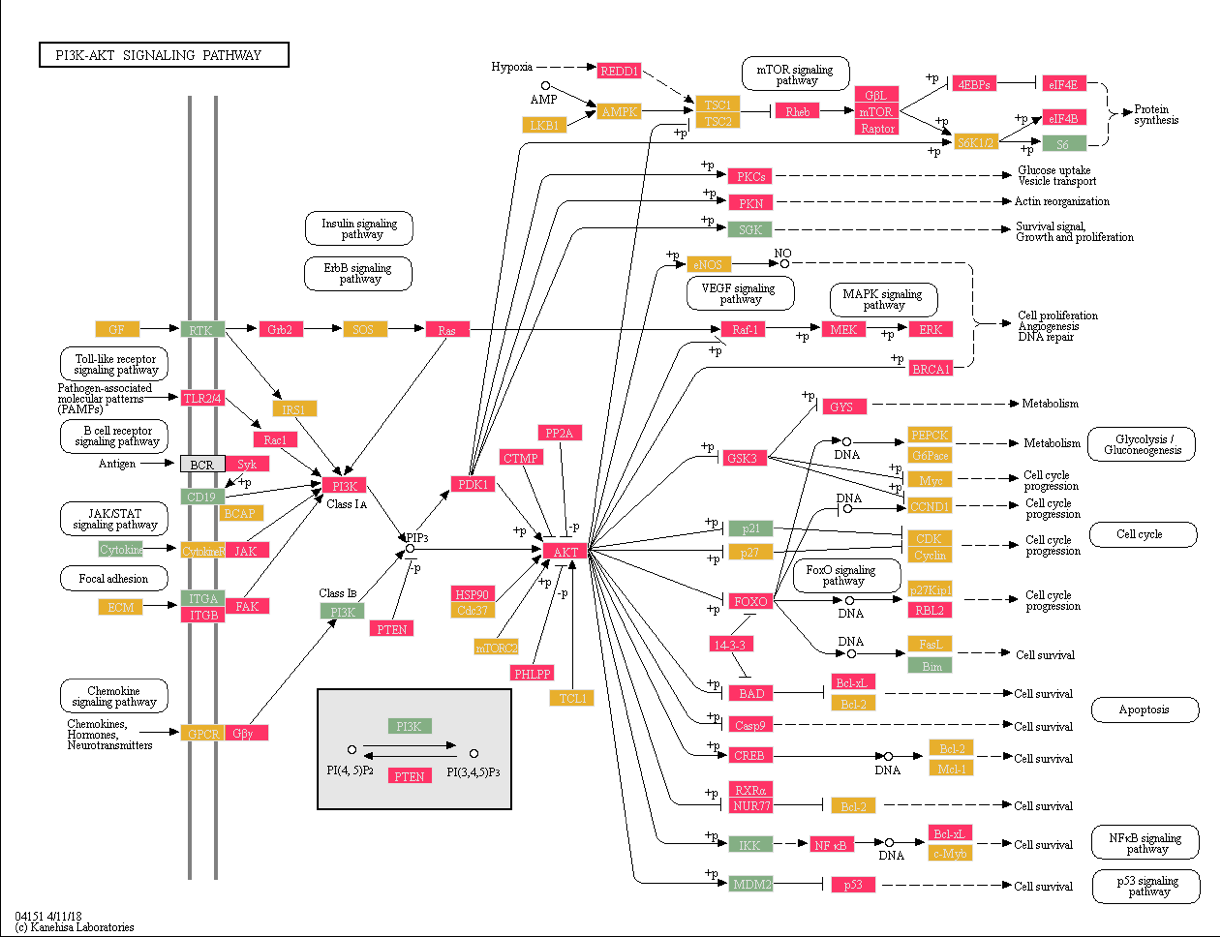

Supplement: S2 Fig — Levels of relative fold change across cell lines (RFC) as indicated: yellow, |RFC| < 0.5; red, RFC < -0.5; green, RFC > 0.5. (TIF) [file pone.0215247.s002.tif]

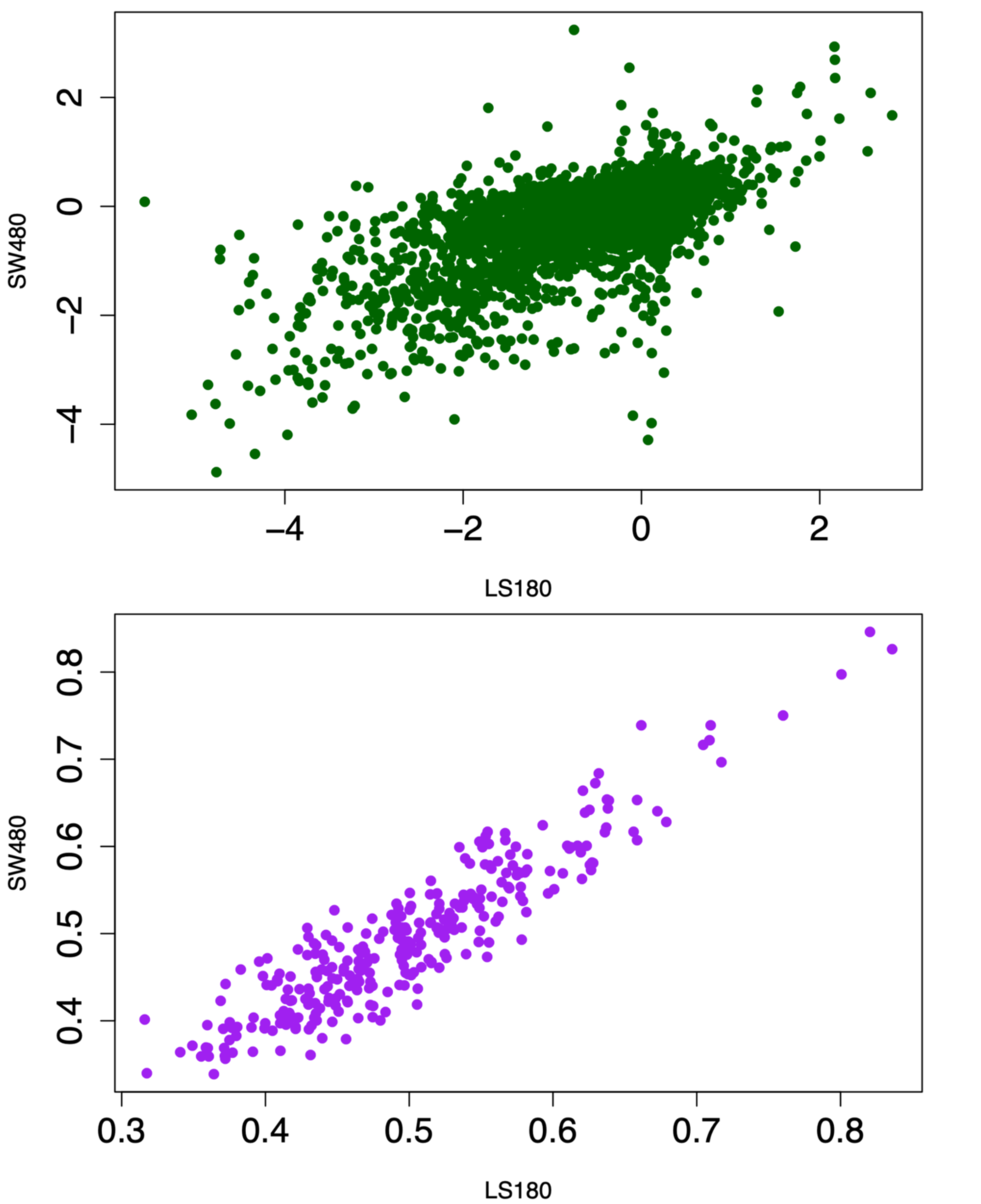

Supplement: S3 Fig — Above, green: correlation of gene expressions in LS180 (horizontal axis) and SW480 (vertical axis); below, purple: correlation of predicted side effect scores in LS180 (horizontal) and SW480 (vertical). (TIF) [file pone.0215247.s003.tif]

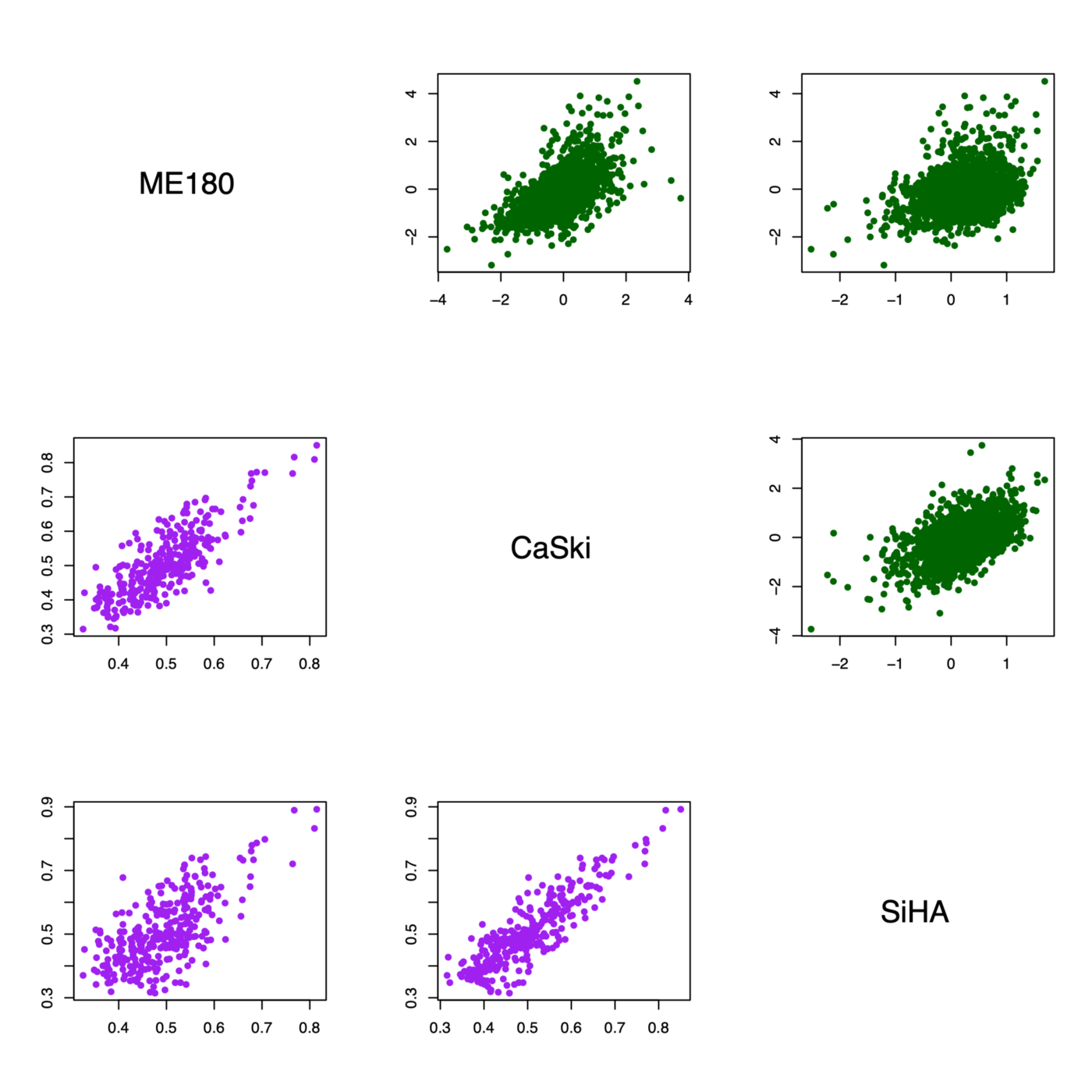

Supplement: S4 Fig — Above diagonal, correlations of expressions (green); below diagonal, pairwise correlation of side effect scores (purple). Cell lines are labelled diagonally across the vertical and horizontal axes. (TIF) [file pone.0215247.s004.tif]

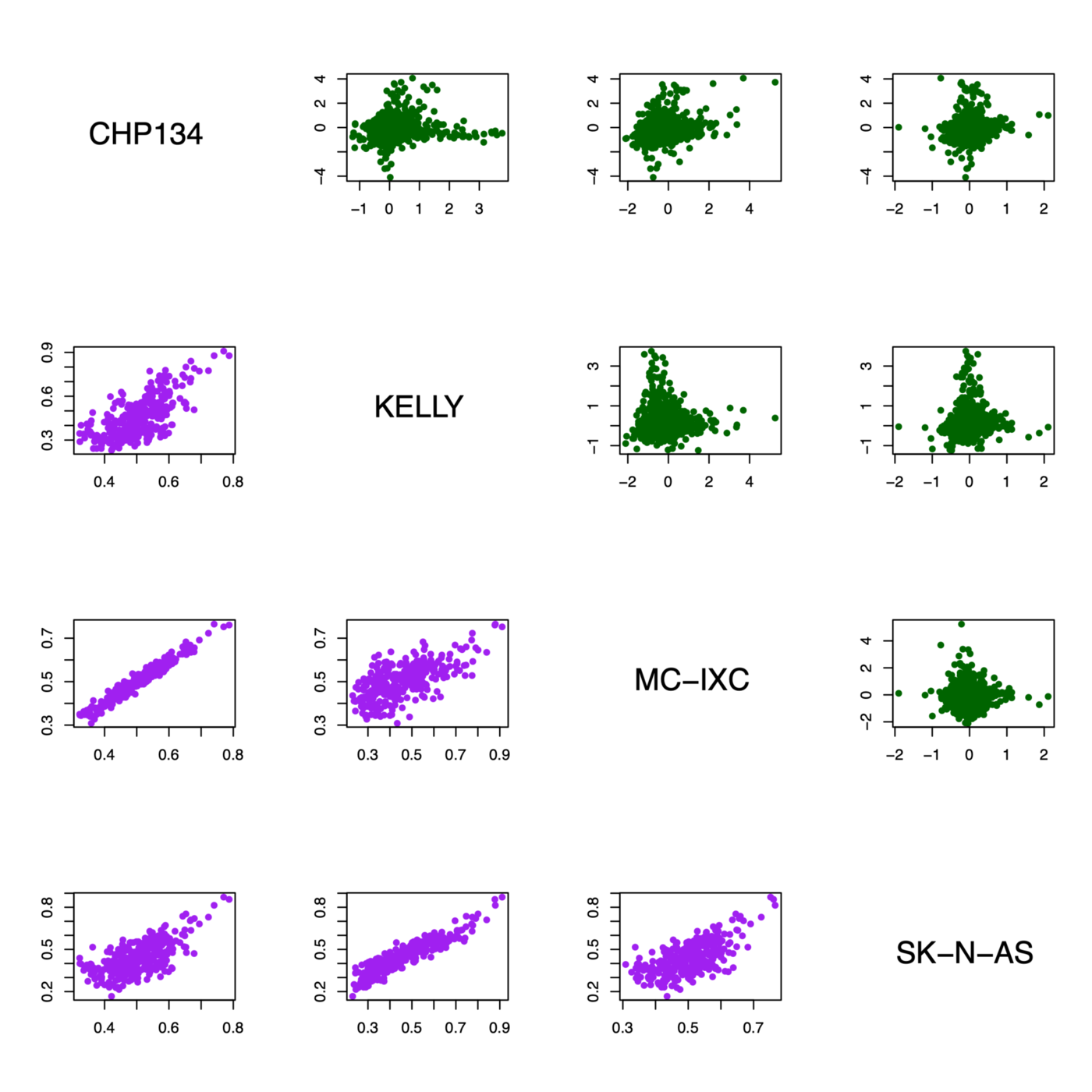

Supplement: S5 Fig — Above diagonal, correlations of expressions (green); below diagonal, pairwise correlation of side effect scores (purple). Cell lines are labelled diagonally across the vertical and horizontal axes. (TIF) [file pone.0215247.s005.tif]

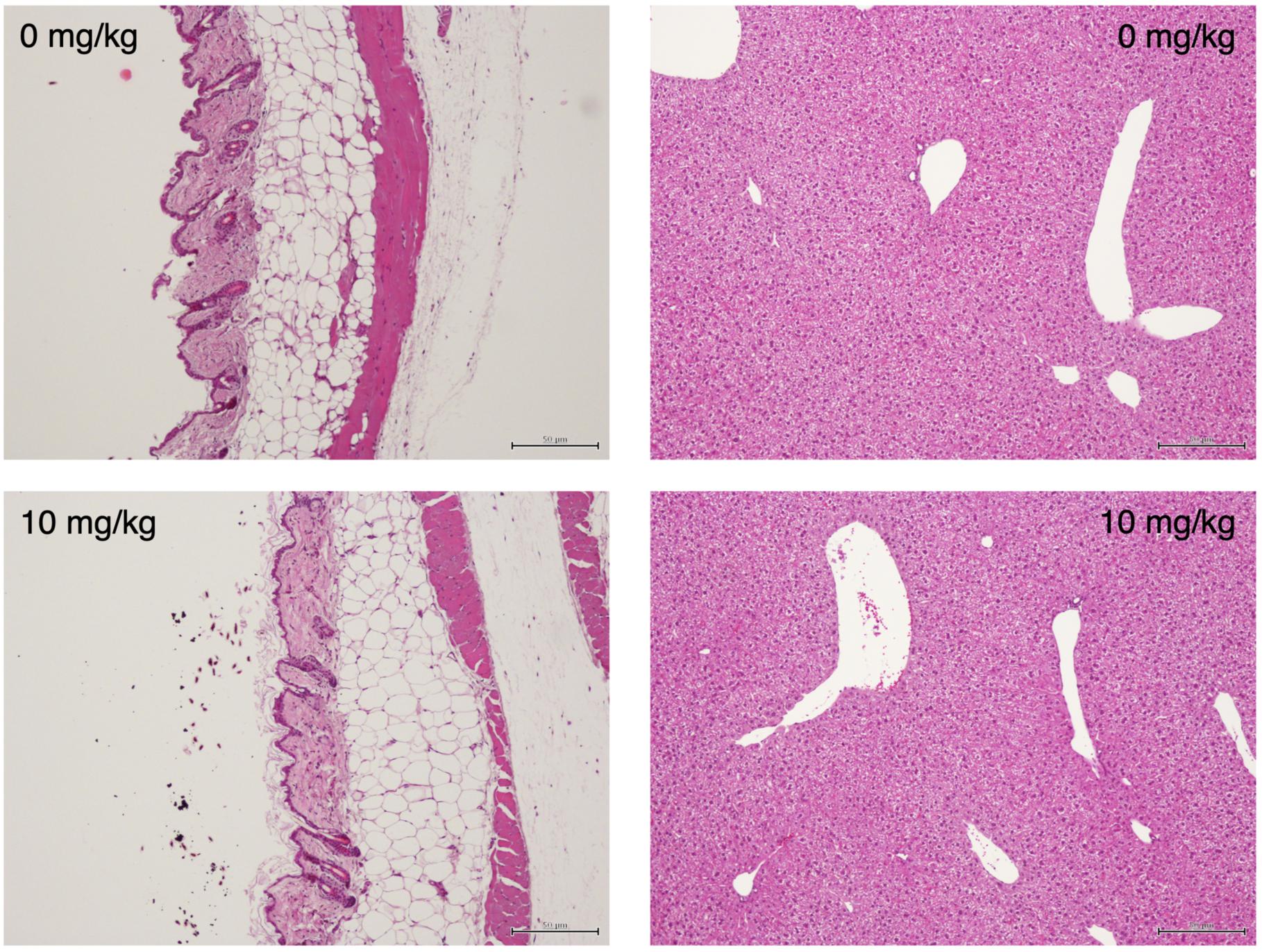

Supplement: S6 Fig — Left half, skin; right half, liver tissue staining. DMSO (top half) is labeled 0 mg/kg; bottom, polyamide 4 at the dose of 10 mg/kg. (TIF) [file pone.0215247.s006.tif]
