# Supplementary material for: Estimating genome-wide off-target effects for pyrrole-imidazole polyamide binding by a pathway-based expression profiling approach
Source: PLoS One. 2019 Apr 9;14(4):e0215247. doi: 10.1371/journal.pone.0215247 (PMC6456183; doi:10.1371/journal.pone.0215247)
Supplement: S1 Table — ATCC, American Type Culture Collection; ECACC, European Collection of Authenticated Cell Cultures; RIKEN, RIKEN BioResource Research Center Cell Bank; NCI, National Cancer Institute Division of Cancer Treatment and Diagnosis Tumor Repository; MEM, Eagle’s Minimum Essential Medium; DMEM, Dulbecco’s Modified Eagle’s Medium; RPMI-1640, Roswell Park Memorial Institute medium 1640; IMDM, Iscove’s Modified Dulbecco’s Medium; FBS, fetal bovine serum; Gln, glutamine (if supplemented). (PDF) [file pone.0215247.s007.pdf]

S1 Table. List of Cells and Culture Conditions. ATCC, American Type Culture Collection; ECACC, European Collection of Authenticated Cell Cultures; RIKEN, RIKEN BioResource Research Center Cell Bank; NCI, National Cancer Institute Division of Cancer Treatment and Diagnosis Tumor Repository; MEM, Eagle's Minimum Essential Medium; DMEM, Dulbecco's Modified Eagle's Medium; RPMI-1640, Roswell Park Memorial Institute medium 1640; IMDM, Iscove's Modified Dulbecco's Medium; FBS, fetal bovine serum; Gln, glutamine (if supplemented).

Cell Line	Source	Medium	FBS [%]	Gln [mM]
LS180	ATCC	MEM	10	
SK-MES-1	ECACC	MEM	10	2
SiHA	ATCC	MEM	10	
SW480	ATCC	DMEM	10	
HT29	ATCC	DMEM	10	
A549	RIKEN	DMEM	10	
HCT116	NCI	DMEM	10	
GP2d	Sigma	DMEM	10	
SK-LU-1	ATCC	DMEM	10	
MIA PaCa-2	ATCC	DMEM	10	
ME180	RIKEN	RPMI-1640	10	
CaSki	RIKEN	RPMI-1640	10	
CHP-134	ECACC	RPMI-1640	10	
Kelly	ECACC	RPMI-1640	10	
HCT15	NCI	RPMI-1640	10	
NCI-H747	ATCC	RPMI-1640	10	
CW-2	RIKEN	RPMI-1640	10	
Colo 205	RIKEN	RPMI-1640	10	
LS513	ATCC	RPMI-1640	10	
LU65	RIKEN	RPMI-1640	10	
SHP-77	ATCC	RPMI-1640	10	
AsPC1	ATCC	RPMI-1640	10	
LoVo	ATCC	Ham's F-12K	10	
MC-IXC	ATCC	1:1 MEM/Ham's F-12	10	
SK-N-AS	ECACC	1:1 MEM/Ham's F-12	10	
RCM-1	JCRB	1:1 RPMI-1640/F-12	10	
KP4	RIKEN	1:1 DMEM/F-12	10	
T84	ATCC	1:1 DMEM/F-12	5	2.5
MCF-10A	ATCC	1:1 DMEM/F-12	10	
Capan-1	ATCC	IMDM	20	
SW837	ATCC	Leibovitz's L-15	10	
SW403	ATCC	Leibovitz's L-15	10	
