# Supplementary material for: Estimating genome-wide off-target effects for pyrrole-imidazole polyamide binding by a pathway-based expression profiling approach
Source: PLoS One. 2019 Apr 9;14(4):e0215247. doi: 10.1371/journal.pone.0215247 (PMC6456183; doi:10.1371/journal.pone.0215247)
Supplement: S2 Table — (PDF) [file pone.0215247.s008.pdf]

S2 Table. Training Expression Datasets for the Side Effect Prediction Model.

Drug	Dataset	Treatment	Control	Probe Set
Acetaminophen	GSE53216	GSM1287778	GSM1287781	GPL13916
Alendronate	GSE63009	GSM1537949	GSM1537946	GPL570
Amiloride	GSE24581	GSM605963	GSM605966	GPL5188
Anagrelide	GSE60621	GSM1483851	GSM1483848	GPL570
Anastrozole	GSE93204	GSM2448486	GSM2448485	GPL6480
Arsenic trioxide	GSE33520	GSM829378	GSM829381	GPL6480
Atorvastatin	GSE63427	GSM1548543	GSM1548544	GPL10558
Azathioprine	GSE60408	GSM1478373	GSM1478420	GPL13667
Celecoxib	GSE11237	GSM283127	GSM283128	GPL8300
Cladribine	GSE60408	GSM1478407	GSM1478420	GPL13667
Cocaine	GSE71939	GSM1847677	GSM1847671	GPL570
Diclofenac	GSE45052	GSM1096649	GSM1096648	GPL6104
Disulfiram	GSE45224	GSM1099424	GSM1099422	GPL13497
Entacapone	GSE85180	GSM2445808	GSM2259766	GPL570
Ephedrine	GSE85871	GSM2286204	GSM2286248	GPL571
Felodipine	GSE60408	GSM1478429	GSM1478420	GPL13667
Finasteride	GSE108998	GSM2927836	GSM2927827	GPL6244
Fluoxetine	GSE83386	GSM2200521	GSM2200520	GPL17077
Flurbiprofen	GSE60408	GSM1478363	GSM1478420	GPL13667
Fluvastatin	GSE59007	GSM1424418	GSM1424415	GPL10558
Furosemide	GSE46909	GSM1140882	GSM1140954	GPL16311
Gemcitabine	GSE61802	GSM1513984	GSM1513985	GPL570
Hexachlorophene	GSE60408	GSM1478435	GSM1478420	GPL13667
Hydralazine	GSE8604	GSM213376	GSM213339	GPL2895
Hydrochlorothiazide	GSE60408	GSM1478411	GSM1478420	GPL13667
Ibuprofen	GSE85180	GSM2445806	GSM2445803	GPL570
Imipramine	GSE72363	GSM1861048	GSM1861047	GPL10904
Indomethacin	GSE45052	GSM1096650	GSM1096648	GPL6104
Itraconazole	GSE60408	GSM1478382	GSM1478420	GPL13667
Lansoprazole	GSE77239	GSM2046439	GSM2046427	GPL570
Leflunomide	GSE24526	GSM604787	GSM604784	GPL6884
Methotrexate	GSE45867	GSM1116958	GSM1116957	GPL570
Methylphenidate	GSE52889	GSM1277540	GSM1277538	GPL570
Miconazole	GSE60408	GSM1478351	GSM1478420	GPL13667
Mycophenolic acid	GSE46909	GSM1140902	GSM1140954	GPL16311
Nefazodone	GSE54254	GSM1311112	GSM1311090	GPL13158
Norepinephrine	GSE64635	GSM1575918	GSM1575910	GPL17077
Nortriptyline	GSE85333	GSM2264872	GSM2264869	GPL22299
Omeprazole	GSE77239	GSM2046433	GSM2046427	GPL570
Pentoxifylline	GSE60408	GSM1478428	GSM1478420	GPL13667

Piroxicam	GSE12860	GSM322615	GSM322609	GPL96
Pravastatin	GSE47458	GSM1149942	GSM1149939	GPL10558
Progesterone	GSE108998	GSM2927833	GSM2927827	GPL6244
Rabeprazole	GSE107162	GSM2861882	GSM2861881	GPL13607
Ribavirin	GSE60408	GSM1478427	GSM1478420	GPL13667
Risedronate	GSE63009	GSM1537949	GSM1537946	GPL570
Rofecoxib	GSE59671	GSM1442224	GSM1442208	GPL571
Rosiglitazone	GSE60408	GSM1478445	GSM1478420	GPL13667
Rosuvastatin	GSE24187	GSM595053	GSM595063	GPL570
Scopolamine	GSE982	GSM15552	GSM15451	GPL96
Simvastatin	GSE51444	GSM1245908	GSM1245907	GPL10558
Sulfasalazine	GSE60408	GSM1478353	GSM1478420	GPL13667
Sulindac	GSE60408	GSM1478376	GSM1478420	GPL13667
Tadalafil	GSE75173	GSM1944595	GSM1944594	GPL15034
Tolcapone	GSE85180	GSM2445809	GSM2445803	GPL570
Topiramate	GSE107015	GSM2859595	GSM2859600	GPL570
Troglitazone	GSE60408	GSM1478562	GSM1478420	GPL13667
Valproic acid	GSE60408	GSM1478611	GSM1478420	GPL13667
