# Supplementary material for: Estimating genome-wide off-target effects for pyrrole-imidazole polyamide binding by a pathway-based expression profiling approach
Source: PLoS One. 2019 Apr 9;14(4):e0215247. doi: 10.1371/journal.pone.0215247 (PMC6456183; doi:10.1371/journal.pone.0215247)
Supplement: S3 Table — Values computed from average tissue-type U133 Plus 2 PIK3CA expressions in GENT as of August 2018; logFC indicates the log ratio of tumor vs. normal expressions for a particular tissue. (PDF) [file pone.0215247.s009.pdf]

S3 Table. Relative Distribution of *PIK3CA* in Various Tumor and Normal Tissues. Values computed from average tissue-type U133 Plus 2 *PIK3CA* expressions in GENT as of August 2018; *logFC* indicates the log ratio of tumor vs. normal expressions for a particular tissue.

Tissue	Tumor	Normal	logFC
Adipose	281.0	566.6	-0.701
Adrenal gland	210.4	220.2	-0.045
Bladder	281.4	359.7	-0.246
Blood	351.7	378.3	-0.073
Brain	428.3	345.6	0.214
Breast	281.8	280.4	0.005
Cervix	359.2	198.2	0.594
Colon	201.7	191.4	0.053
Endometrium	258.0	206.7	0.222
Esophagus	157.9	210.8	-0.289
Head & neck	321.5	160.4	0.696
Kidney	315.0	240.0	0.272
Liver	234.7	158.7	0.391
Lung	286.3	280.2	0.022
Ovary	353.0	186.1	0.640
Pancreas	229.6	257.0	-0.113
Prostate	236.9	259.3	-0.090
Skin	220.2	303.9	-0.322
Small intestine	316.5	169.5	0.624
Stomach	237.0	181.8	0.265
Testis	221.2	201.9	0.092
Thyroid	232.7	165.0	0.344
Uterus	282.1	567.4	-0.699
Vagina	346.0	237.0	0.378
Vulva	293.9	235.6	0.221
