# Supplementary material for: Estimating genome-wide off-target effects for pyrrole-imidazole polyamide binding by a pathway-based expression profiling approach
Source: PLoS One. 2019 Apr 9;14(4):e0215247. doi: 10.1371/journal.pone.0215247 (PMC6456183; doi:10.1371/journal.pone.0215247)
Supplement: S4 Table — KRAS G12, mutation status for codon 12 in KRAS; GNA14, expression of GNA14 in U133A datasets compiled by the Gene Expression Across Normal and Tumor Tissue Database. Class, classification of cell lines into “Off-Target” or “G12D/V” in Fig 4A. (PDF) [file pone.0215247.s010.pdf]

S4 Table. Summary of Cytotoxicity of Polyamide 4 and *GNA14* Expressions in Various Cell Lines. *KRAS* G12, mutation status for codon 12 in *KRAS*; *GNA14*, expression of *GNA14* in U133A datasets compiled by the Gene Expression Across Normal and Tumor Tissue Database. *Class*, classification of cell lines into "Off-Target" or "G12D/V" in Fig 4A.

Cell Line	Tissue	*KRAS* G12	IC_{50} [nM]	*GNA14*	Class
HCT-15	Colorectal	WT	478.2	66	Off-Target
HT29	Colorectal	WT	318.0	133	Off-Target
NCI-H747	Colorectal	WT	289.0	7	Off-Target
CW-2	Colorectal	WT	159.9	18	Off-Target
LoVo	Colorectal	WT	68.7	7	Off-Target
T84	Colorectal	WT	53.4	5	Off-Target
Colo 205	Colorectal	WT	45.6	57	Off-Target
SW837	Colorectal	G12C	32.0	21	Off-Target
HCT116	Colorectal	WT	10.9	16	Off-Target
GP2d	Colorectal	G12D	48.2	39	G12D/V
RCM-1	Colorectal	G12V	30.4	1	G12D/V
LS513	Colorectal	G12D	22.0	26	G12D/V
SW403	Colorectal	G12V	7.1	13	G12D/V
MCF10A	Breast	WT	1.4	27	Off-Target
PC-9	Lung	WT	74.7	89	Off-Target
SK-MES-1	Lung	WT	48.6	15	Off-Target
LU65	Lung	G12C	23.9	42	Off-Target
A549	Lung	G12S	12.0	38	Off-Target
SK-LU-1	Lung	G12D	28.8	35	G12D/V
SHP-77	Lung	G12D	12.0	3	G12D/V
MIA PaCa-2	Pancreas	WT	49.9	6	Off-Target
RCM-1	Pancreas	G12V	30.4	1	G12D/V
Capan-1	Pancreas	G12V	20.2	30	G12D/V
AsPC-1	Pancreas	G12D	19.1	16	G12D/V
KP4	Pancreas	G12D	3.1	1	G12D/V
