# Supplementary material for: Estimating genome-wide off-target effects for pyrrole-imidazole polyamide binding by a pathway-based expression profiling approach
Source: PLoS One. 2019 Apr 9;14(4):e0215247. doi: 10.1371/journal.pone.0215247 (PMC6456183; doi:10.1371/journal.pone.0215247)
Supplement: S5 Table — Predicted scores are on a scale of 0–1; synd., syndrome; inc., increase; dec., decrease; did., disorder. The table is illustrated as a heatmap in Fig 3B. (PDF) [file pone.0215247.s011.pdf]

S5 Table. Common Potential Side Effects of Polyamides 1 – 3 from Metabolic Gene Expression Profiling. Predicted scores are on a scale of 0 – 1; *synd.*, syndrome; *inc.*, increase; *dec.*, decrease; *did.*, disorder. The table is illustrated as a heatmap in Fig 3B.

**Polyamide**<br>Cell Line	**1**<br>LS-180	**1**<br>SW-480	**2**<br>SiHA	**2**<br>ME-180	**2**<br>CaSki	**3**<br>KELLY	**3**<br>MC-IXC	**3**<br>SK-N-AS	**3**<br>CHP-134
Abdominal discomfort	0.442	0.498	0.497	0.525	0.488	0.474	0.493	0.491	0.465
Abdominal pain	0.659	0.653	0.643	0.602	0.665	0.674	0.628	0.606	0.642
Acute coronary *synd.*	0.569	0.554	0.597	0.508	0.600	0.550	0.526	0.512	0.542
Agitation	0.514	0.471	0.513	0.549	0.521	0.514	0.538	0.475	0.526
Agranulocytosis	0.562	0.519	0.454	0.446	0.546	0.516	0.487	0.452	0.501
ALT *inc.*	0.521	0.461	0.470	0.526	0.531	0.465	0.535	0.424	0.548
Alopecia	0.627	0.573	0.584	0.543	0.654	0.656	0.563	0.551	0.583
Anaemia	0.500	0.491	0.568	0.407	0.558	0.554	0.432	0.454	0.433
Anaphylactic shock	0.535	0.530	0.567	0.422	0.566	0.630	0.430	0.501	0.453
Angina pectoris	0.535	0.599	0.654	0.544	0.534	0.477	0.531	0.510	0.518
Angioedema	0.441	0.470	0.511	0.441	0.464	0.557	0.428	0.492	0.451
Angiopathy	0.401	0.472	0.508	0.478	0.509	0.478	0.427	0.484	0.409
Anorexia	0.659	0.607	0.686	0.543	0.680	0.738	0.575	0.558	0.601
Anxiety	0.597	0.546	0.718	0.538	0.626	0.659	0.523	0.551	0.547
Arrhythmia	0.538	0.538	0.519	0.440	0.578	0.513	0.490	0.448	0.490
Arthralgia	0.549	0.606	0.673	0.539	0.593	0.570	0.464	0.536	0.456
Arthritis	0.547	0.534	0.576	0.489	0.506	0.498	0.512	0.444	0.516
AST *inc.*	0.506	0.419	0.401	0.545	0.505	0.469	0.577	0.449	0.583
Asthenia	0.717	0.697	0.722	0.764	0.768	0.775	0.724	0.732	0.723
Asthma	0.507	0.512	0.570	0.516	0.483	0.438	0.509	0.428	0.509
Back pain	0.530	0.519	0.543	0.611	0.511	0.566	0.584	0.578	0.576
Blood and lymphatic system *did.*	0.492	0.521	0.487	0.530	0.511	0.529	0.515	0.548	0.519

Body temperature *inc.*	0.610	0.601	0.654	0.511	0.639	0.694	0.536	0.575	0.545
Breast *did.*	0.501	0.532	0.502	0.546	0.494	0.447	0.534	0.482	0.509
Bronchitis	0.474	0.517	0.579	0.478	0.473	0.449	0.464	0.425	0.481
Cardiac *did.*	0.570	0.591	0.557	0.540	0.560	0.532	0.596	0.542	0.610
Cerebrovascular accident	0.526	0.505	0.614	0.537	0.576	0.469	0.492	0.422	0.495
Chest pain	0.557	0.575	0.639	0.552	0.597	0.636	0.556	0.624	0.542
Chills	0.581	0.570	0.668	0.502	0.620	0.538	0.478	0.484	0.497
Confusional state	0.549	0.530	0.582	0.587	0.569	0.637	0.546	0.566	0.565
Conjunctivitis	0.501	0.547	0.632	0.553	0.553	0.490	0.507	0.513	0.507
Connective tissue *did.*	0.500	0.527	0.515	0.509	0.521	0.508	0.530	0.556	0.546
Constipation	0.621	0.664	0.739	0.654	0.671	0.706	0.592	0.630	0.619
Convulsion	0.508	0.501	0.532	0.539	0.531	0.508	0.499	0.489	0.529
Cough	0.577	0.570	0.624	0.539	0.580	0.565	0.544	0.525	0.540
Appetite *dec.*	0.656	0.617	0.692	0.581	0.692	0.717	0.553	0.580	0.586
Depression	0.528	0.524	0.537	0.509	0.512	0.463	0.515	0.488	0.509
Dermatitis	0.821	0.846	0.889	0.768	0.816	0.911	0.752	0.874	0.770
Diarrhoea	0.710	0.739	0.780	0.679	0.747	0.791	0.656	0.722	0.679
Discomfort	0.636	0.617	0.744	0.582	0.697	0.774	0.528	0.631	0.541
Disturbance in sexual arousal	0.499	0.476	0.490	0.548	0.467	0.460	0.511	0.526	0.524
Dizziness	0.661	0.739	0.734	0.683	0.676	0.748	0.660	0.737	0.643
Dry mouth	0.519	0.506	0.556	0.552	0.538	0.557	0.577	0.521	0.567
Dysgeusia	0.578	0.493	0.493	0.561	0.542	0.587	0.601	0.446	0.587
Dyspepsia	0.638	0.654	0.707	0.580	0.625	0.673	0.571	0.591	0.575
Dyspnoea	0.593	0.624	0.740	0.553	0.620	0.645	0.526	0.585	0.547
Dysuria	0.567	0.616	0.644	0.518	0.574	0.478	0.529	0.463	0.557
Ear and labyrinth *did.*	0.410	0.454	0.453	0.521	0.423	0.419	0.460	0.456	0.449
Eosinophilia	0.550	0.550	0.652	0.484	0.635	0.511	0.535	0.409	0.524
Epistaxis	0.549	0.540	0.545	0.544	0.541	0.500	0.554	0.480	0.555

Erectile dysfunction	0.607	0.569	0.556	0.656	0.598	0.561	0.666	0.599	0.650
Eructation	0.506	0.495	0.515	0.528	0.492	0.425	0.512	0.404	0.496
Erythema multiforme	0.538	0.544	0.595	0.494	0.583	0.645	0.518	0.564	0.538
Eye *did.*	0.435	0.456	0.418	0.554	0.473	0.501	0.567	0.549	0.530
Eye pain	0.465	0.445	0.430	0.527	0.453	0.414	0.514	0.412	0.506
Face oedema	0.495	0.469	0.458	0.506	0.496	0.480	0.495	0.484	0.485
Fatigue	0.627	0.581	0.650	0.675	0.638	0.697	0.669	0.705	0.667
Feeling abnormal	0.637	0.622	0.682	0.560	0.686	0.700	0.572	0.595	0.593
Flatulence	0.529	0.513	0.467	0.526	0.483	0.467	0.536	0.428	0.533
Flushing	0.519	0.546	0.594	0.538	0.561	0.547	0.523	0.557	0.499
Gastritis	0.545	0.542	0.524	0.559	0.527	0.502	0.576	0.487	0.563
Gastroenteritis	0.521	0.501	0.493	0.575	0.520	0.474	0.588	0.490	0.569
Gastrointestinal *did.*	0.551	0.599	0.681	0.561	0.570	0.599	0.547	0.581	0.527
Gastrointestinal pain	0.622	0.639	0.619	0.590	0.623	0.670	0.604	0.561	0.622
Gynaecomastia	0.560	0.514	0.505	0.583	0.559	0.515	0.545	0.536	0.526
Haematuria	0.613	0.599	0.599	0.524	0.616	0.523	0.597	0.504	0.605
Haemoglobin	0.542	0.580	0.623	0.552	0.559	0.503	0.573	0.481	0.590
Haemorrhage	0.562	0.583	0.610	0.591	0.551	0.530	0.561	0.483	0.592
Hallucination	0.582	0.525	0.579	0.537	0.567	0.492	0.547	0.465	0.581
Headache	0.836	0.827	0.893	0.815	0.851	0.878	0.762	0.857	0.787
Hepatic enzyme *inc.*	0.564	0.559	0.465	0.534	0.521	0.445	0.576	0.480	0.577
Hepatic failure	0.628	0.581	0.481	0.586	0.573	0.556	0.649	0.521	0.648
Hepatic function abnormal	0.464	0.435	0.484	0.479	0.487	0.505	0.466	0.550	0.480
Hepatic necrosis	0.540	0.477	0.457	0.578	0.497	0.449	0.586	0.463	0.566
Hepatitis	0.612	0.597	0.601	0.573	0.604	0.640	0.573	0.526	0.554
Hepatobiliary disease	0.525	0.477	0.471	0.541	0.520	0.548	0.539	0.531	0.546
Hepatocellular injury	0.525	0.496	0.480	0.452	0.481	0.420	0.536	0.419	0.506
Hot flush	0.531	0.504	0.463	0.567	0.506	0.518	0.625	0.478	0.654

Hyperhidrosis	0.619	0.594	0.652	0.587	0.646	0.706	0.584	0.637	0.576
Hypersensitivity	0.629	0.673	0.687	0.597	0.665	0.778	0.595	0.672	0.590
Hypertension	0.673	0.641	0.648	0.614	0.657	0.729	0.649	0.641	0.649
Hypoaesthesia	0.568	0.570	0.608	0.659	0.631	0.551	0.683	0.518	0.653
Hypotension	0.493	0.501	0.574	0.479	0.530	0.546	0.436	0.493	0.453
Ill-defined *did.*	0.617	0.601	0.716	0.538	0.660	0.742	0.529	0.581	0.571
Immune system *did.*	0.469	0.479	0.483	0.483	0.508	0.535	0.502	0.557	0.505
Infection	0.625	0.642	0.619	0.583	0.641	0.637	0.624	0.540	0.601
Infestation	0.521	0.535	0.541	0.518	0.535	0.460	0.495	0.518	0.471
Infestation NOS	0.545	0.540	0.527	0.501	0.519	0.483	0.516	0.496	0.512
Influenza	0.576	0.571	0.605	0.535	0.584	0.507	0.557	0.412	0.572
Insomnia	0.679	0.628	0.681	0.676	0.732	0.722	0.652	0.636	0.669
Jaundice	0.519	0.507	0.506	0.501	0.565	0.518	0.511	0.439	0.500
Leukopenia	0.601	0.551	0.591	0.436	0.594	0.655	0.555	0.513	0.534
Libido *dec.*	0.452	0.485	0.505	0.531	0.468	0.428	0.463	0.502	0.457
Liver function test abnormal	0.555	0.603	0.573	0.556	0.555	0.509	0.583	0.464	0.595
Loss of consciousness	0.537	0.530	0.577	0.581	0.561	0.542	0.583	0.546	0.576
Lymphadenopathy	0.493	0.529	0.633	0.462	0.523	0.438	0.474	0.415	0.480
Malaise	0.623	0.601	0.705	0.536	0.654	0.707	0.556	0.599	0.567
Malnutrition	0.467	0.458	0.457	0.499	0.494	0.457	0.516	0.494	0.512
Mediastinal *did.*	0.519	0.546	0.561	0.568	0.536	0.529	0.543	0.563	0.556
Menopausal symptoms	0.556	0.490	0.445	0.541	0.537	0.509	0.637	0.472	0.679
Mental *did.*	0.555	0.617	0.624	0.593	0.550	0.524	0.583	0.569	0.564
Migraine	0.457	0.507	0.511	0.465	0.463	0.422	0.469	0.427	0.478
Mouth ulceration	0.515	0.513	0.492	0.501	0.505	0.417	0.530	0.417	0.515
Muscle spasms	0.488	0.522	0.630	0.489	0.500	0.522	0.467	0.506	0.472
Muscular weakness	0.515	0.513	0.475	0.538	0.527	0.498	0.541	0.463	0.540
Musculoskeletal discomfort	0.582	0.591	0.648	0.573	0.600	0.595	0.553	0.581	0.542

Myalgia	0.582	0.573	0.661	0.570	0.603	0.621	0.534	0.550	0.541
Myocardial infarction	0.577	0.543	0.577	0.518	0.592	0.526	0.516	0.521	0.513
Nausea	0.760	0.750	0.762	0.678	0.769	0.772	0.691	0.682	0.695
Nervous system *did.*	0.553	0.612	0.560	0.596	0.577	0.583	0.588	0.598	0.586
Nervousness	0.537	0.507	0.505	0.532	0.558	0.580	0.569	0.541	0.583
Neuropathy peripheral	0.626	0.579	0.611	0.551	0.616	0.606	0.578	0.505	0.601
Neutropenia	0.490	0.504	0.499	0.445	0.474	0.437	0.443	0.447	0.459
Oedema	0.519	0.523	0.572	0.506	0.584	0.598	0.423	0.512	0.419
Oedema peripheral	0.578	0.554	0.605	0.574	0.579	0.587	0.568	0.587	0.582
Pain	0.579	0.538	0.598	0.623	0.589	0.622	0.597	0.577	0.603
Pain in extremity	0.423	0.482	0.482	0.593	0.428	0.430	0.535	0.548	0.489
Palpitations	0.539	0.586	0.653	0.490	0.599	0.539	0.495	0.550	0.482
Pancytopenia	0.570	0.552	0.571	0.496	0.630	0.593	0.519	0.494	0.533
Paraesthesia	0.638	0.644	0.733	0.660	0.693	0.635	0.611	0.611	0.587
Petechiae	0.548	0.491	0.528	0.530	0.606	0.497	0.483	0.467	0.492
Pharyngitis	0.598	0.572	0.621	0.608	0.575	0.586	0.628	0.550	0.635
Photosensitivity reaction	0.443	0.457	0.458	0.430	0.485	0.517	0.416	0.420	0.428
Pneumonia	0.531	0.518	0.462	0.493	0.534	0.467	0.549	0.423	0.554
Pollakiuria	0.497	0.476	0.477	0.519	0.504	0.461	0.501	0.435	0.496
Pruritus	0.709	0.722	0.798	0.706	0.771	0.801	0.646	0.755	0.653
Purpura	0.521	0.505	0.556	0.501	0.558	0.563	0.489	0.482	0.522
Rash	0.801	0.797	0.832	0.810	0.809	0.880	0.766	0.815	0.741
Renal failure	0.515	0.545	0.562	0.491	0.549	0.469	0.472	0.491	0.478
Renal impairment	0.505	0.479	0.493	0.507	0.534	0.433	0.448	0.465	0.433
Rhinitis	0.572	0.578	0.644	0.534	0.554	0.551	0.562	0.480	0.556
Shock	0.498	0.504	0.509	0.581	0.510	0.559	0.555	0.603	0.548
Sinusitis	0.565	0.536	0.501	0.521	0.493	0.500	0.568	0.461	0.567
Skin *did.*	0.574	0.600	0.574	0.588	0.583	0.618	0.590	0.594	0.590

Somnolence	0.530	0.507	0.483	0.624	0.586	0.602	0.555	0.621	0.549
Stevens-Johnson *synd.*	0.553	0.580	0.560	0.480	0.603	0.608	0.511	0.525	0.536
Stomatitis	0.620	0.563	0.551	0.563	0.632	0.555	0.602	0.478	0.611
Sweating	0.521	0.529	0.594	0.500	0.559	0.615	0.455	0.568	0.458
Sweating *inc.*	0.526	0.472	0.501	0.506	0.493	0.489	0.563	0.461	0.562
Syncope	0.557	0.542	0.547	0.562	0.580	0.515	0.581	0.510	0.571
Tachycardia	0.497	0.507	0.592	0.464	0.562	0.543	0.466	0.511	0.475
Tension	0.552	0.520	0.467	0.500	0.566	0.600	0.582	0.542	0.577
Thrombocytopenia	0.639	0.653	0.610	0.542	0.671	0.654	0.550	0.530	0.578
Tinnitus	0.494	0.520	0.532	0.549	0.560	0.495	0.519	0.465	0.518
Toxic epidermal necrolysis	0.549	0.503	0.457	0.448	0.557	0.547	0.500	0.479	0.523
Tremor	0.556	0.579	0.644	0.552	0.580	0.628	0.538	0.579	0.552
Unspecified skin and subcutaneous tissue *did.*	0.575	0.567	0.573	0.558	0.584	0.564	0.550	0.588	0.552
Upper respiratory tract infection	0.429	0.507	0.584	0.469	0.505	0.460	0.453	0.504	0.458
Urethral *did.*	0.465	0.482	0.456	0.516	0.485	0.495	0.531	0.530	0.521
Urinary tract *did.*	0.497	0.491	0.467	0.516	0.504	0.498	0.524	0.530	0.538
Urinary tract infection	0.567	0.607	0.595	0.507	0.546	0.516	0.555	0.476	0.574
Urticaria	0.632	0.684	0.734	0.571	0.657	0.746	0.577	0.662	0.586
Vascular purpura	0.490	0.504	0.542	0.520	0.554	0.558	0.489	0.493	0.514
Vertigo	0.515	0.560	0.526	0.477	0.512	0.488	0.499	0.470	0.521
Vision blurred	0.532	0.534	0.502	0.487	0.538	0.567	0.485	0.501	0.444
Visual impairment	0.468	0.485	0.505	0.481	0.491	0.439	0.509	0.448	0.501
Vomiting	0.705	0.716	0.786	0.689	0.772	0.841	0.636	0.712	0.668
Weight *dec.*	0.530	0.506	0.552	0.531	0.506	0.473	0.609	0.423	0.593
Weight *inc.*	0.554	0.473	0.493	0.558	0.570	0.554	0.610	0.504	0.613
