# Supplementary material for: Estimating genome-wide off-target effects for pyrrole-imidazole polyamide binding by a pathway-based expression profiling approach
Source: PLoS One. 2019 Apr 9;14(4):e0215247. doi: 10.1371/journal.pone.0215247 (PMC6456183; doi:10.1371/journal.pone.0215247)
Supplement: S6 Table — RPR, mean relative percentile rank of specified gene in all participating pathways; p0, t-test p-value under H0: RPR = 0.5000; p1, t-test p-value under H0: RPROff-Target = RPRTarget. Significance evaluated against a predefined threshold of p < 0.05 (log10 p < -1.3010); N/S, not significant; N/A, not evaluated; [a]number of pathways evaluated for KRAS (“foreground”): 68; number of background pathways: 244. (PDF) [file pone.0215247.s012.pdf]

S6 Table. Potential Off-Targets of Polyamide 1 in LS180 Colorectal Cancer Cell Line. *RPR*, mean relative percentile rank of specified gene in all participating pathways; p_0, *t*-test *p*-value under H_0: $RPR = 0.5000$; p_1, *t*-test *p*-value under H_0: $RPR_{Off\text{-}Target} = RPR_{Target}$. Significance evaluated against a predefined threshold of $p < 0.05$ ($\log_{10} p < -1.3010$); *N/S*, not significant; *N/A*, not evaluated; [a]number of pathways evaluated for *KRAS* ("foreground"): 68; number of background pathways: 244.

Symbol	Class	RPR	$\log_{10} p_0$	$\log_{10} p_1$	Significance
KRAS	Target	0.3084[a]	-11.7893	N/A	N/A
EEA1	Off-Target	0.0315	-6.2952	-8.9997	Significant
GNA14	Off-Target	0.1875	-2.2760	-2.3353	Significant
GNAL	Off-Target	0.2724	-1.7158	-0.2102	N/S
GRIA4	Off-Target	0.2071	-4.3569	-3.6233	Significant
NSF	Off-Target	0.2008	-2.6420	-1.9864	Significant
NT5C2	Off-Target	0.2936	-2.1976	-0.1448	N/S
PDE4B	Off-Target	0.1673	-3.3558	-3.0680	Significant
PDE4D	Off-Target	0.0752	-5.4828	-7.0500	Significant
POLR3A	Off-Target	0.0942	-4.7175	-8.9344	Significant
POLR3B	Off-Target	0.0248	-7.5277	-36.0436	Significant
RFC1	Off-Target	0.1144	-4.0167	-5.3723	Significant
RFC3	Off-Target	0.2948	-1.9417	-0.1309	N/S
RORA	Off-Target	0.0958	-3.0342	-6.0166	Significant

[a]number of pathways evaluated for KRAS ("foreground"): 68; number of background pathways: 244.
